# IL7R Is Correlated With Immune Cell Infiltration in the Tumor Microenvironment of Lung Adenocarcinoma

**DOI:** 10.3389/fphar.2022.857289

**Published:** 2022-02-21

**Authors:** Xin Wang, Shujian Chang, Teng Wang, Ruirong Wu, Zebo Huang, Junjie Sun, Jingjing Liu, Yan Yu, Yong Mao

**Affiliations:** ^1^ Department of Medical Oncology, Affiliated Hospital of Jiangnan University, Wuxi, China; ^2^ Department of Medical Oncology, Harbin Medical University Cancer Hospital, Harbin, China

**Keywords:** IL7R, tumor immunological microenvironment, tumor infiltrating lymphocytes, lung adenocarcinoma, prognosis

## Abstract

Tumor microenvironment plays an important role in the development, progression, and prognosis of lung adenocarcinoma. Exploring new biomarkers based on the immune microenvironment of lung adenocarcinoma can effectively predict the prognosis and provide effective clinical treatment. In this study, we used the ESTIMATE algorithm to score the immune and stromal components in lung adenocarcinoma data downloaded from the TCGA database. The result showed that the immune/stromal score was associated with clinical features and prognosis of lung adenocarcinoma patients. Interleukin-7 receptor (IL7R) is an important prognostic biomarker identified by intersection analysis of protein-protein interaction networks and Cox regression survival analysis. According to TCGA and Oncomine database analysis, IL7R expression in adenocarcinoma tissues was significantly lower than that in normal lung tissues and was further verified in clinical tissue samples. Survival analysis showed IL7R was an independent prognostic factor of lung adenocarcinoma. IL7R expression was positively correlated with the overall survival and progression-free survival of lung adenocarcinoma patients and negatively correlated with tumor size. Our results suggest that IL7R inhibits tumor growth by regulating the proportion of immune infiltrating cells in the tumor immune microenvironment. IL7R could be a beneficial prognostic marker in patients with lung adenocarcinoma and has great potential in immune therapy.

## Introduction

Lung cancer is the leading cause of cancer-related death with increasing morbidity and mortality over the years. Non-small cell lung cancer (NSCLC) accounts for about 85% of lung cancers, and lung adenocarcinoma (LUAD) is a common pathological type of NSCLC with a 5 year survival rate of less than 20% (Chen et al., 2015; DeSantis et al., 2016; Bray et al., 2018; Siegel et al., 2021). Tumor immunotherapy, such as immune checkpoint blockade (ICB), has dramatically changed the landscape of cancer treatment and improved the survival of various cancer patients (Lambrechts et al., 2018; Zappasodi et al., 2018). Since not all tumor patients respond to immunotherapy, searching for effective therapeutic targets and prognostic biomarkers for individualized treatment has become an urgent need.

Previous studies have suggested that the positive response of immunotherapy usually depends on the dynamic interaction between tumor cells and immune modulators in the tumor microenvironment (TME) (Dougan and Dranoff, 2009a). The inhibitory effects and heterogeneity of TME have a great influence on the occurrence and development of tumors and the efficacy of immunotherapy (Dougan and Dranoff, 2009b). The TME is composed of vascular, extracellular matrix (ECM), cancer-associated fibroblasts (CAFs) and tumor-infiltrating lymphocytes (TILs). The tumor immune microenvironment plays an important role in the development and progression of primary lung cancer (Lavin et al., 2017). In this study, the immune and stromal components in the TME of lung adenocarcinoma were scored by the ESTIMATE algorithm. We found that the immune and stromal scores were related to the pathological molecular subtypes, clinical stages, and survival prognosis of LUAD patients. Based on different immune/stromal scores, we screened out differential expression genes related to the immune microenvironment and identified prognostic genes associated with survival. IL7R was confirmed to be associated with prognosis and immune cell infiltration in LUAD patients. IL7R may be a prognostic factor and a potential therapeutic target for immunotherapy for patients with LUAD.

## Materials and Methods

### Ethics Statement

The pathological tissue sections used in this study were approved by the Ethics Committee of Jiangnan university affiliated hospital. Each patient signed a consent form for participating in the study.

### Data Acquisition and Analysis

The mRNA expression data and clinical information of lung adenocarcinoma were obtained from the TCGA and GEO database (GSE68465). There are three molecular subtypes of lung adenocarcinoma: bronchoid, magnoid, and squamoid (Hayes et al., 2006). The stromal and immune scores were calculated by the ESTIMATE algorithm using the TCGA data (Yoshihara et al., 2013). The survminer package was used to analyze the relationship between stromal score or immune score and overall survival of LUAD patients. The optimal cut-off points were calculated by smoothHR (Smooth Hazard Ratio Curves Taking a Reference Value) algorithm. The expression of IL7R in lung adenocarcinoma tissues and normal tissues was analyzed by TCGA and Oncomine database. CIBERSORT computational method was applied for estimating the TIL abundance profile in all tumor samples (Chen et al., 2018). The relationship between IL7R and TIL was evaluated by the Timer website (Li et al., 2017).

### Differential Expression Genes and Functional Enrichment

Differential expression genes (DEGs) were screened out using the limma package of R language, Fold change (FC) >1 and *p* < 0.05 (Li et al., 2017). Heatmap was generated using ClustVis (Ritchie et al., 2015). Functional enrichment analysis on DEGs was conducted by the web tool DAVID. False discovery rate (FDR) < 0.05 (Metsalu and Vilo, 2015). The KEGG pathway function of IL7R was enriched and analyzed by GSEA (Huang et al., 2009).

### Protein-Protein Interaction Network Construction and Cox Survival Analysis

The Protein-Protein Interaction (PPI) network was constructed by the STRING database (Subramanian et al., 2005) and visualized with the Cytoscape software (Szklarczyk et al., 2015). Nodes were selected with an interaction relationship greater than 0.5 ((Shannon et al., 2003)). The Cox survival analysis was analyzed using the software package survival to screen prognostic genes (*p* < 0.05).

### Patients and Tissue Samples

In this study,40 cases of LUAD and 35 cases of adjacent normal tissues were collected from LUAD patients who underwent surgery and then received care and follow-up in our hospital between the period of July 2014 and August 2016.

### Cell Culture

A panel of LUAD cell lines H1975, H1650, H1299, A549, PC9, H661, and the normal lung epithelial cell line HBE were used for *in vitro* validation. All cells were purchased from the ATCC. The PC9 cell line was maintained in DMEM medium (Gibco, NY, United States), and the other cells were maintained in 1,640 medium (Gibco, NY, United States), both mediums containing 10% FBS. All cells were incubated in 5% CO_2_ air at 37°C.

### Immunohistochemical Analysis

The paraffin sections were dewaxed with xylene, immersed in the EDTA antigen extraction buffer for antigen repair, blocked with 3% hydrogen peroxide, incubated with rabbit anti-IL7R polyclonal antibody (1:200; ABclonal; A13503) overnight. Tissue sections were incubated with the secondary antibody (Zhongshan Golden Bridge, PV-6001), then stained with DAB.

Immunohistochemical (IHC) staining results were evaluated by two experienced pathologists who were blinded to all original data. IL7R staining is mainly located in the cytoplasm of bronchial epithelium or alveolar epithelium of lung tissue, and also in the cytoplasm of tumor cells. In the IL7R staining evaluation, a semiquantitative scoring method was used to assess the IHC staining result, which included the staining intensity score and score of staining density. We calculated the intensity of staining (0, no staining; 1, low-intensity staining; 2, moderate-intensity staining; 3, strong intensity staining) and the staining density (1, 0–10%; 2, 11–50%; 3, 51–100%). The total IHC score (staining intensity score × staining density score) ranged from 0 to 9. The IHC score <4 was defined as IL7R low expression and the IHC score ≥4 was defined as IL7R high expression.

### RT-qPCR

Total RNA was extracted and reverse transcription was performed based on the manufacturer’s protocol. A NanoDrop 2000 (Thermo, MA, United States) was used to measure the RNA concentration. RT-qPCR was performed with an ABI Illumina instrument (Steponeplus, United States) using SYBRGreen (Roche, Swiss). Semi-quantitative analysis of the mRNA expression level was quantified by the 2^−ΔΔCt^ method.

### Western Blot

Cells were harvested and lysed with RIPA lysis solution (Solarbio, China) supplemented with protease and phosphorylase inhibitors. The concentration of cell protein was determined by the BCA assay kit (Beyotime, China) and equal amounts of proteins were resolved by SDS-PAGE gel and transferred to the PVDF membrane. Afterward, the primary and secondary antibodies were added for incubation. Finally, the membranes were washed with PBST and visualized with ECL detection reagent (Beyotime, China). The primary antibodies were as follows: IL7R (1:1,000; ABclonal; A13503) and β-actin (1:1,000; Zhongshan Golden Bridge; TA-09).

### Statistical Analysis

Discrete and continuous data are presented as a count with proportion and mean (± standard deviation) or median (interquartile range [IQR]), respectively, with standard methods for group comparison. Comparison between two groups was performed by Students t-test. One way-ANOVA was used for comparison in multiple groups. The association between IL7R expression and clinicopathological parameters was performed by the chi-squared test. Survival curves were drawn based on the Kaplan-Meier method. *p* < 0.05 was considered significantly different. All statistical analyses were performed by using the R language (version 3.5.3).

## Results

### Immune Scores and Stromal Scores Are Significantly Associated With LUAD Molecular Subtypes, Clinical Stage, and Survival

**FIGURE 1:**
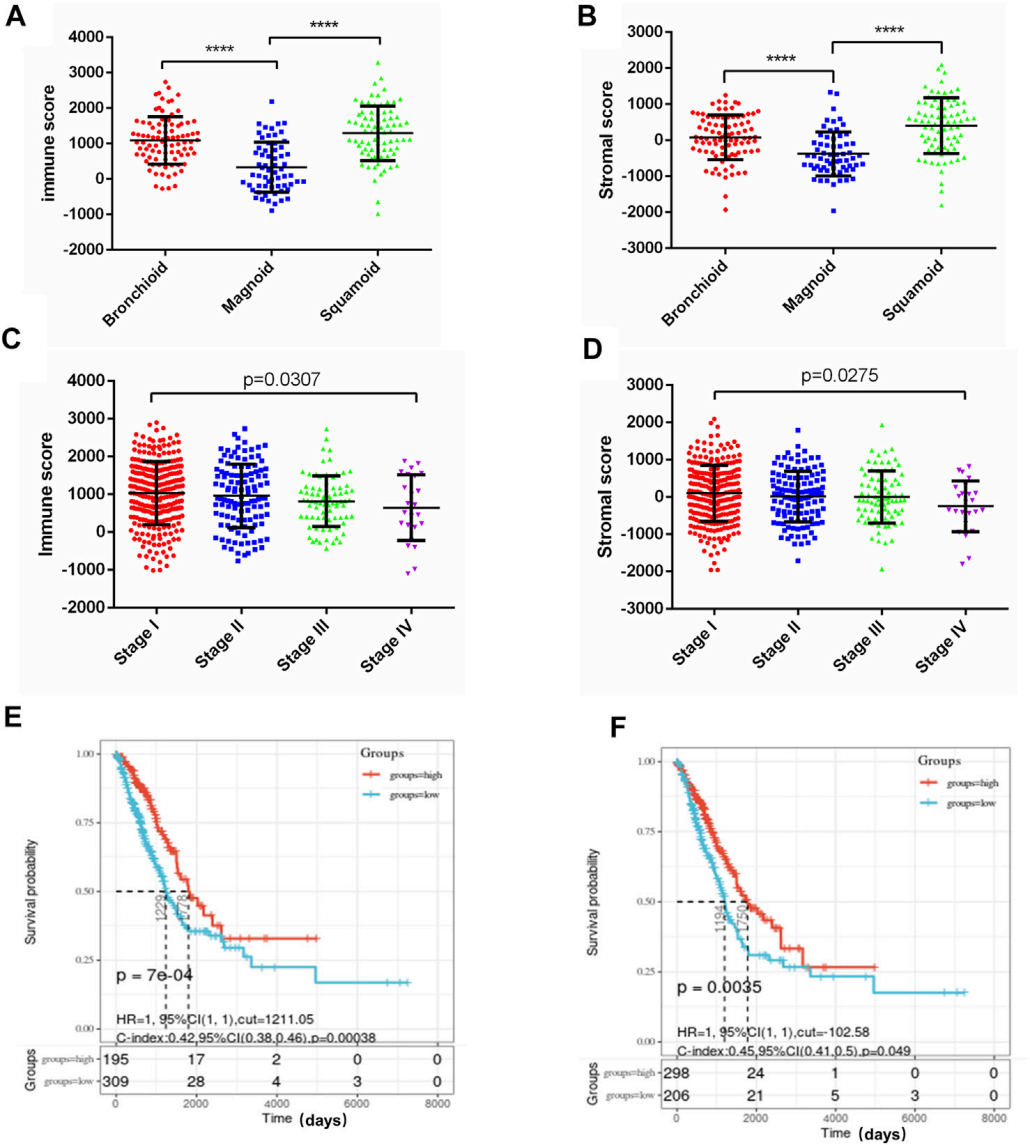
Immune scores and stromal scores are associated with LUAD subtypes, clinical stage, and survival. **(A)** Distribution of immune scores in LUAD molecular subtypes. Box‐plot shows a significant association between molecular subtypes and the level of immune scores (*n* = 516, *p* < 0.001). **(B)** Distribution of stromal scores of LUAD molecular subtypes. Box‐plot shows a significant association between LUAD molecular subtypes and the level of stromal scores (*n* = 516, *p* < 0.001). **(C)** Distribution of immune scores for LUAD clinical stage. Box‐plot shows a significant association between the clinical stage and the level of immune scores (*n* = 516, *p* = 0.0307). **(D)** Distribution of stromal scores for LUAD clinical stage. Box‐plot shows a significant association between the clinical stage and the level of immune scores (*n* = 516, *p* = 0.0275). **(E)** The Kaplan‐Meier survival curve showed the overall survival of the high score group is longer than the low score group, *p* = 7e-04. **(F)** LUAD cases were divided into two groups based on their stromal scores, the overall survival of the high score group is longer than the low score group, *p* = 0.035. **** *p* < 0.0001.

We downloaded the data of lung adenocarcinoma mRNA expression and clinical profiles from the TCGA database. In this study, we obtained information about 516 LUAD patients from the TCGA database and calculated the immune score and stromal score of each sample by ESTIMATE algorithm. The range of immune score was from −13,355.85 to 3,286.67, and the range of stromal score was from −1959.31 to 2098.77. We found that the immune score and stromal score were significantly associated with LUAD molecular subtypes, clinical stage, and survival. The squamoid subtype had the highest average immune score, followed by the bronchoid subtype. Patients with the magnoid subtype had the lowest immune scores, *p* < 0.0001 (Figure 1A). The trend of the stromal score was consistent with the immune score, with squamoid subtype having the highest score, magnoid having the lowest score, and bronchoid subtype having the score in the middle, *p* < 0.0001 (Figure 1B). In addition, the immune score and stromal score were decreased with the elevated clinical stage of lung adenocarcinoma. The immune/stromal score of stage I patients was significantly higher than that of stage IV patients (Figures 1C,D). Patients with LUAD were grouped into low-and high-immune/stromal score groups based on the cut-off value for immune scores (1,211.05) or stromal scores (102.58). Survival analysis showed that LUAD patients with higher immune or stromal scores had significantly better overall survival than those with lower scores (Figures 1E,F).

### Screening Differential Genes Related to Tumor Immune Microenvironment in Lung Adenocarcinoma

**FIGURE 2:**
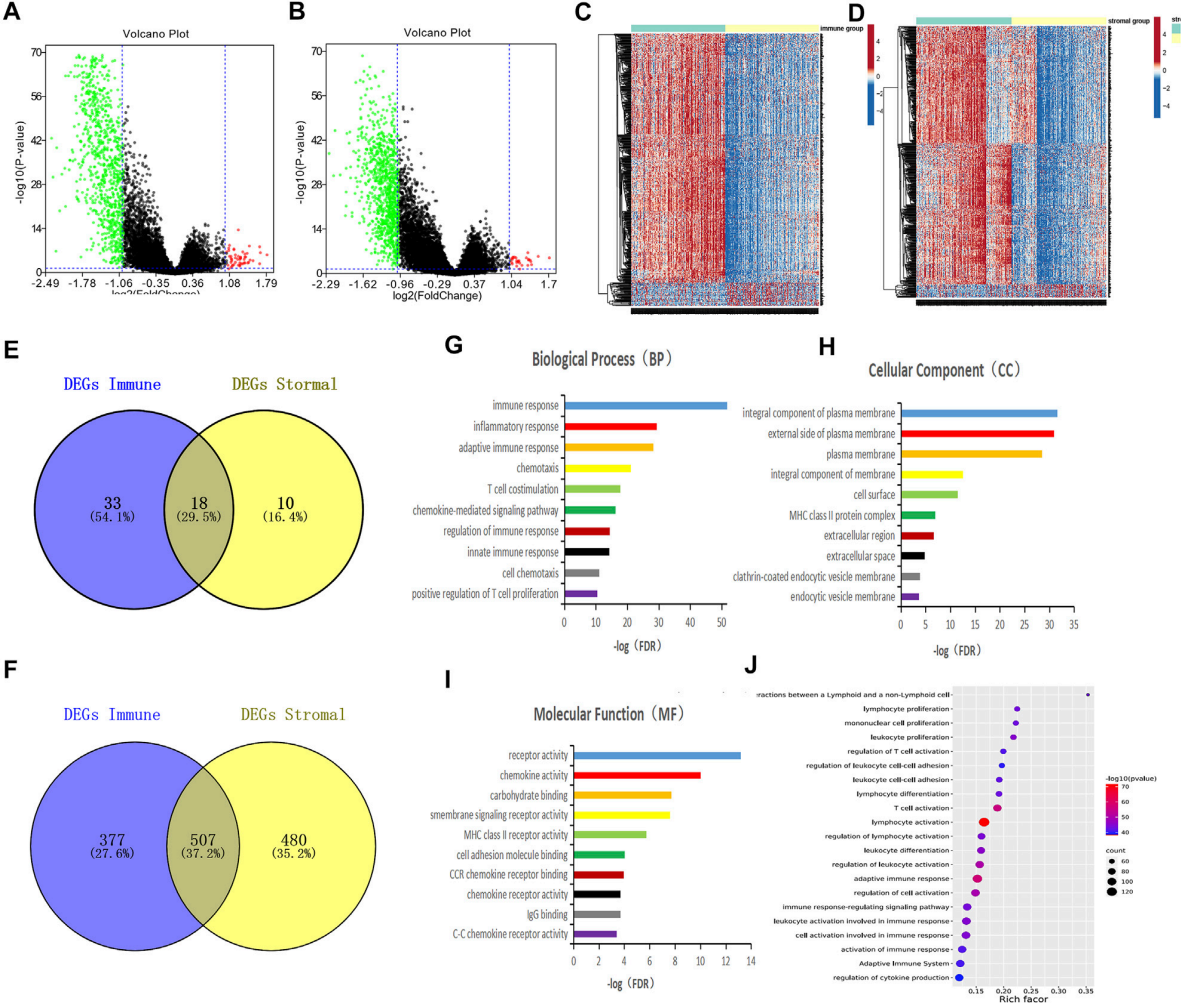
Screening differential genes related to tumor immune microenvironment in lung adenocarcinoma. **(A,B)** Patients with LUAD were grouped according to immune/stromal scores, volcano plots were drawn using Limma package of R language, |log2FC|>1, *p* < 0.05. Genes with high expression are shown in red, low expression is shown in green, genes with the same expression level are in black. **(C,D)** Heatmap of the DEGs of immune/stromal scores of top half (high score) vs bottom half (low score). *p* < 0.05, FC > 1) **(E,F)** Venn diagrams showing the number of commonly upregulated **(E)** or downregulated **(F)** DEGs in stromal and immune score groups. **(G–I)** Top 10 GO terms. False discovery rate (FDR) of GO analysis was acquired from DAVID functional annotation tool, *p* < 0.05. **(J)** Bubble plots showed the annotation of the DEGs KEGG pathway, *p* < 0.05.

To identify genes that play important roles in the TME of LUAD, we evaluated the mRNA sequencing data of 516 LUAD patients in the TCGA database which were divided into high and low score groups according to immune/stromal scores. The differential expression genes (DEGs) were screened out by the Limma package of R language, the results were shown in the Volcano plots (Figures 2A,B). There were 51 up-regulated genes and 884 down-regulated genes in the high immune score group compared with the low score group. Based on stromal scores, 28 genes were up-regulated and 987 genes were down-regulated in the high stromal score group (|log2FC|>1, *p* < 0.05). The heatmap showed distinct expression profiles of the DEGs in LUAD patients according to the immune/stromal scores (Figures 2C,D). A total of 18 up-regulated genes and 507 down-regulated genes were screened as DEGs by drawing a Venn diagram for intersection (Figures 2E,F). Then, we performed GO functional enrichment analysis on 507 down-regulated genes. The result showed the top 10 GO terms of the biological process included immune response, inflammatory response, adaptive immune response, chemotaxis, T cell costimulation, Chemokine-mediated signaling pathway, regulation of immune response, innate immune response, cell chemotaxis, and positive regulation of T cell proliferation (Figure 2G); MHC class II protein complex was shown in the top Go terms of CC(Figure 2H); and receptor activity, chemokine activity, MHC class II receptor activity, IgG binding were indicated in the top GO terms of MF (Figure 2I). The GO enrichment function of the KEGG pathway showed the DEGs were significantly related to the immune response pathway (Figure 2J).

### PPI Network Was Constructed to Screen the Key Genes of Immune Function

**FIGURE 3:**
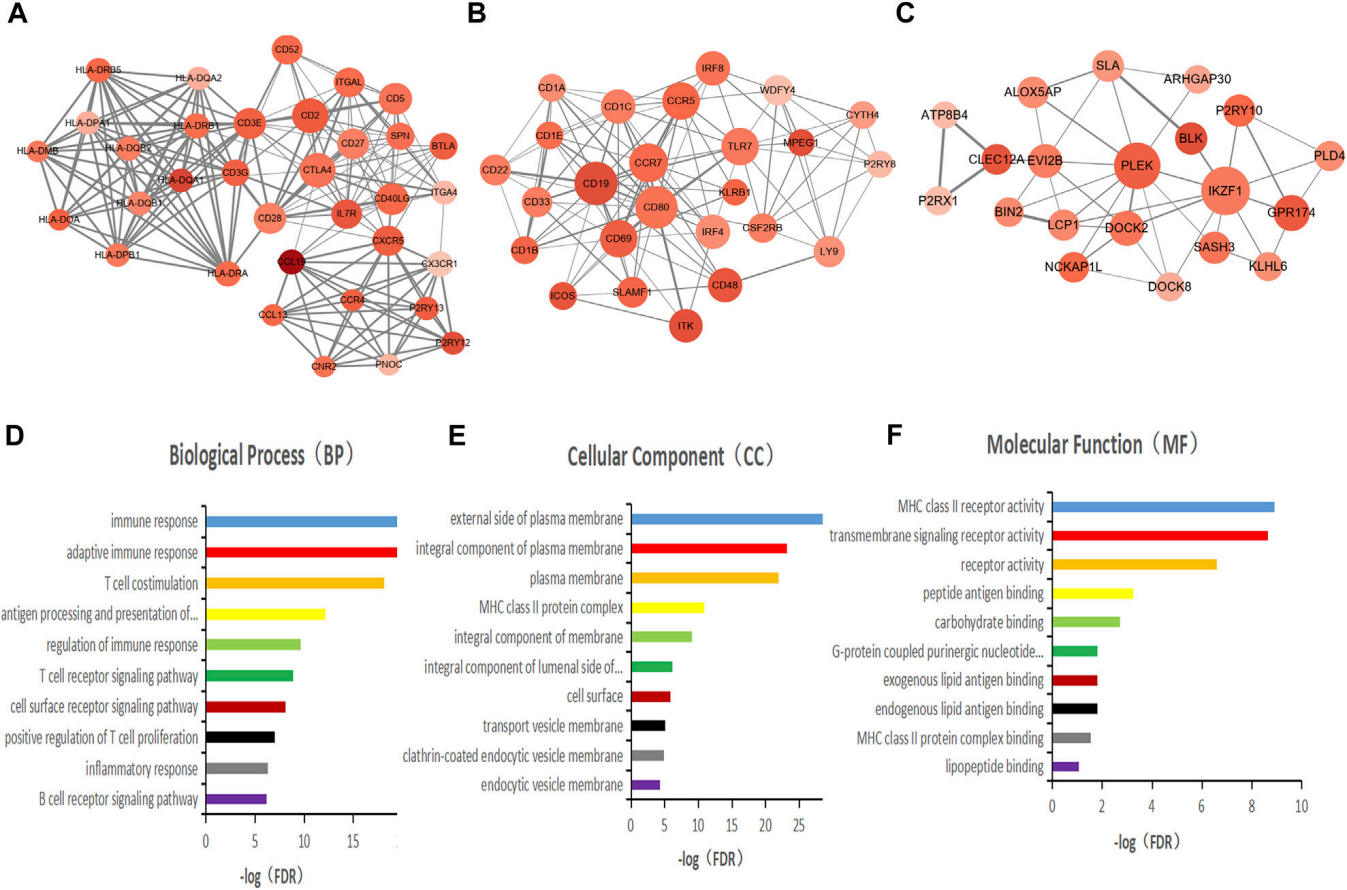
PPI network was constructed to screen the key genes of immune function. **(A–C)** Interaction network constructed with the nodes with interaction confidence value > 0.5. The color of the nodes in the PPI network reflects the log(FC) value of the Z value of gene expression, and the size of the nodes represents the number of proteins interacting with the specified protein. **(D–F)** Top 10 GO terms of the mode gene in figure A. False discovery rate (FDR) of GO analysis was acquired from DAVID functional annotation tool, *p* < 0.05.

To better understand the interactions of the DEGs, we constructed protein-protein interaction (PPI) networks using the STRING online tool. The network consisted of eight modules, 221 nodes, and 1,604 edges. We used Cytoscape software to analyze the PPI network and screened out the most important three modules for further analysis (Figures 3A–C). GO functional enrichment analysis was conducted on the genes in the three modules, and we found that only the first module of the gene set was functionally enriched in the immune function, including immune response, adaptive immune response, T cell costimulation, antigen processing and presentation, regulation of immune response, T cell receptor signaling pathway, cell surface receptor signaling pathway, positive regulation of T cell proliferation, inflammatory response, and B cell receptor signaling pathway (Figures 3D–F).

### Screening Prognostic Genes for Lung Adenocarcinoma

**FIGURE 4:**
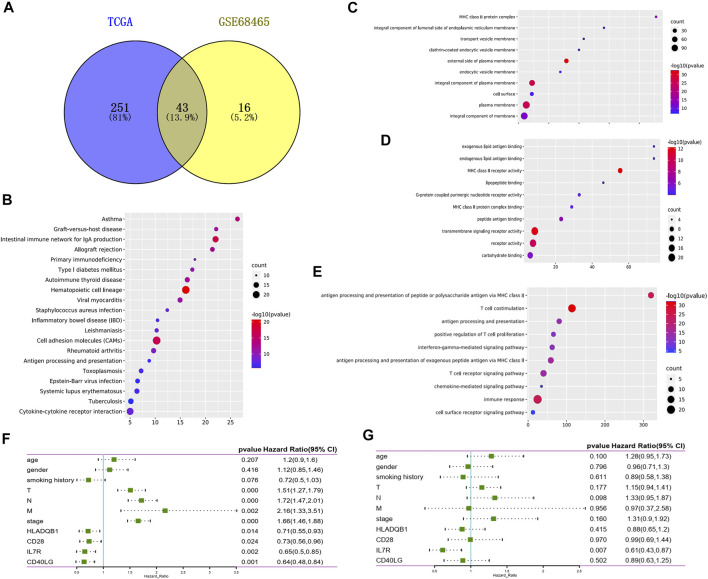
Screening prognostic genes for lung adenocarcinoma. **(A)** Venn plot showed the prognostic gene shared by the TCGA and GSE68465 dataset in univariate COX analysis. **(B)** Bubble plots showed the annotation of the prognostic gene in KEGG pathway. **(C–E)** Top 10 GO terms of the prognostic gene. False discovery rate (FDR) of GO analysis was acquired from the DAVID functional annotation tool, *p* < 0.05. **(F)** Univariate Cox survival analysis of immune microenvironment related prognostic genes based on TCGA lung adenocarcinoma data. **(G)** Variate Cox survival analysis of immune microenvironment related prognostic genes based on TCGA lung adenocarcinoma data.

To explore the potential role of DEGs in the overall survival of LUAD, we performed Cox survival analysis on 507 DEGs. The results showed 294 genes were associated with the OS in patients with LUAD by log-rank test, *p* < 0.05. We also downloaded the GSE68465 data set from the GEO database and screened 59 genes related to the prognosis of LUAD by Cox survival analysis. A total of 43 DEGs were determined to be related to LUAD prognosis after the intersection analysis of the above two datasets by the Venn diagram (Figure4A). GO functional enrichment analysis of these 43 genes showed a significant correlation with immune response, including immune cell activation, signal transduction, and antigen-antibody response (Figures 4B–E). By comparing 43 prognostic genes with 35 immune-related genes, we identified six immune microenvironment-related genes that could predict prognosis, including CD27, IL7R, CD40LG, CD28, CD2, and HLA-DQB1. We then downloaded a list of the immune genes from the Import website, which contains 2,483 immune genes symbols. Four prognostic immune genes were identified by comparing the list. According to the Cox survival analysis of the four genes, the univariate survival analysis result showed that tumor stage (*p* < 0.001), tumor size (*p* < 0.001), lymph node metastasis (*p* < 0.001), distant metastasis (*p* = 0.002), as well as the expression of IL7R (*p* = 0.002), CD40LG (*p* = 0.001), CD28(*p* = 0.024), and HLA-DQB1(*p* = 0.014) all affected the prognosis of LUAD patients (Figure 4F). Multivariate Cox analysis showed that Figure 4F only IL7R expression was an independent prognostic factor affecting the prognosis of lung adenocarcinoma, *p* = 0.007 (Figure 4G).

### Relationship Between IL7R Expression and Clinical Features and Survival in Patients With Lung Adenocarcinoma

**FIGURE 5:**
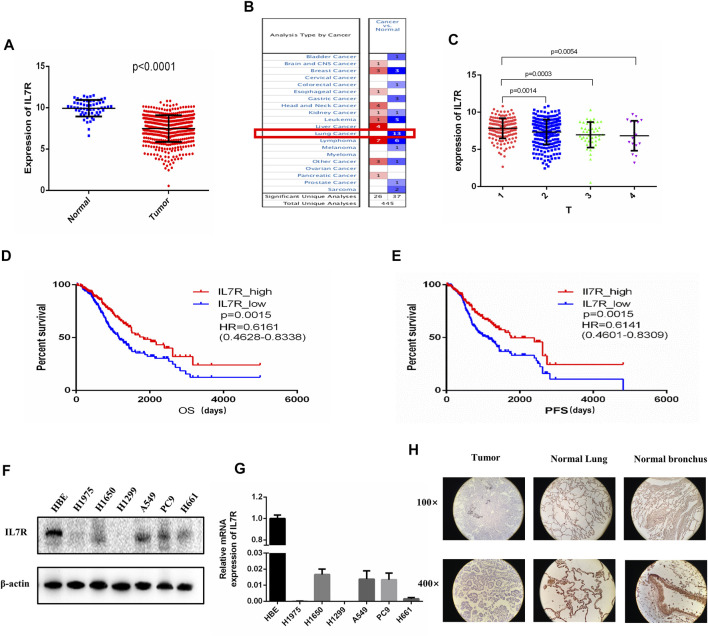
The expression of IL7R in lung adenocarcinoma tissues was lower than that in normal tissues and correlated with tumor size and patient survival. **(A)** IL7R expression in lung adenocarcinoma tissue was significantly lower than that in normal lung tissue based on TCGA data, *p* < 0.001. **(B)** The Oncomine database showed that the expression level of IL7R in lung cancer tissues in 13 data sets was lower than that in normal tissues. **(C)** The expression level of IL7R was negatively correlated with tumor size. **(D)** Kaplan-Meier survival curves showed the LUAD patients with high IL7R expression had better overall survival than those with low IL7R expression, *p* = 0.0015. **(E)** Patients with high IL7R expression also had better progression-free survival than those with low IL7R expression, *p* = 0.0015. **(F)**The expression of IL7R in normal lung epithelial cell line HBE was higher than that in lung adenocarcinoma cell lines assayed by Western blot and **(G)** qRT-PCR. **(H)** Immunohistochemical staining showed IL7R was expressed at low level in LUAD tumors (left) compared with para-carcinoma tissue (lung tissue, middle; bronchus, right), (×100 and×400, respectively).

To clarify the relationship between IL7R and the clinical characteristics of LUAD patients, we analyzed the TCGA data and found that the expression level of IL7R in LUAD tissues was significantly lower than that in normal lung tissues, *p* < 0.001 (Figure 5A). The Oncomine datasets showed the expression of IL7R in lung cancer tissues was decreased in all 13 datasets (Figure 5B). The detailed analysis data were shown in Table 1. Then, we analyzed the relationship between IL7R and clinical features, and the survival of patients with lung adenocarcinoma was analyzed using datasets from TCGA. The expression of IL7R was correlated with the tumor size, the IL7R expression in the T1 stage was significantly higher than in other T stages (Figure 5C). We also found that the LUAD patients with high IL7R expression had significantly better overall survival (OS) and progression-free survival (PFS) than those with low IL7R expression (Figures 5D,E). Further verification was conducted in 40 tissue samples from LUAD patients and 35 cases of adjacent normal tissues, and it was found that the expression of IL7R was negative in lung adenocarcinoma tissues and positive in adjacent normal lung tissues. In lung adenocarcinoma tissue samples, the expression of IL7R is mainly located in the cytoplasm of tumor cells, and most of them are negative or weakly positive (Figure 5F). In addition, we analyzed the expression of IL7R in lung adenocarcinoma tissue samples with different growth patterns. The tissues of 40 LUAD patients included 20 cases of acinar adenocarcinoma (ACI). 15 cases of solid adenocarcinoma with mucin production (SPA), 3 cases were micropapillary predominant adenocarcinoma (MPA) and 2 cases were lepidic predominant adenocarcinoma (LPA). The result showed there was no correlation between the expression of IL7R and the growth pattern of lung adenocarcinoma (*p* = 0.075). We also found that the expression of IL7R in normal lung epithelial cell line was higher than that in lung adenocarcinoma cell lines (Figures 5G,H).

**TABLE 1:** Transcription expression of IL7R between LUAD and normal lung tissues (Oncomine).

Types of LUAD vs. Lung	Fold change	*p*-value	*t*-test	Ref
Lung adenocarcinoma	−6.208	1.5E-6	−6.419	Bhattacharjee Lung
Lung adenocarcinoma	−2.816	1.9E-7	−6.159	Stearman Lung
Lung adenocarcinoma	−2.278	7.41E-4	−4.914	Garber Lung
Lung adenocarcinoma	−2.420	5.7E-8	−6.126	Su Lung
Lung adenocarcinoma	−2.646	1.17E-15	−9.377	Landi Lung
Lung adenocarcinoma	−2.406	1.40E-11	−7.896	Hou Lung
Lung adenocarcinoma	−2.252	1.38E-10	−6.925	Selamat Lung
Lung adenocarcinoma	−1.976	1.77E-4	−4.686	Beer Lung
Lung adenocarcinoma	−1.833	1.25E-5	−5.188	Okayama Lung

### IL7R is Associated With Tumor Microenvironmental Status of Lung Adenocarcinoma

**FIGURE 6:**
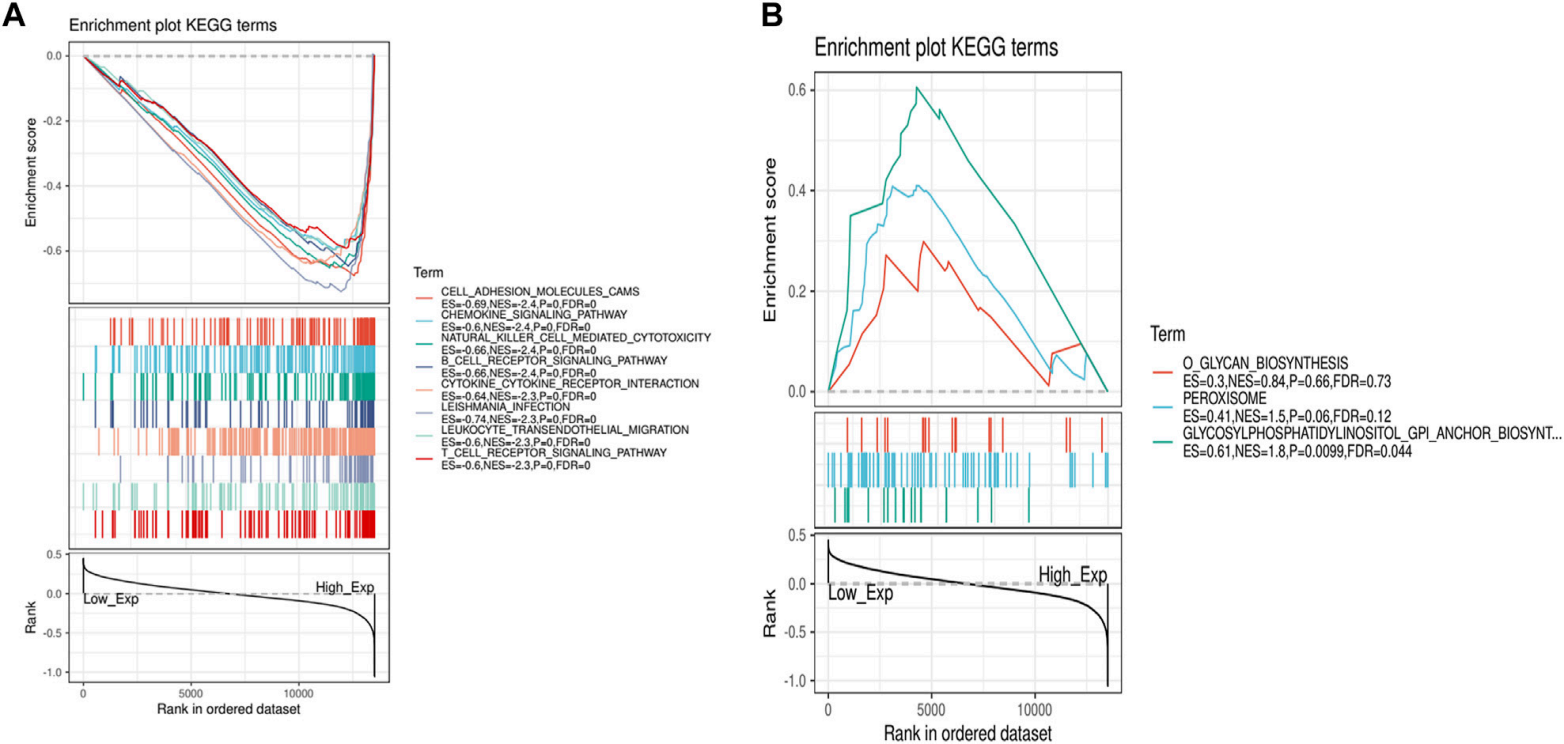
GSEA for samples with high IL7R expression and low expression. **(A)** The enriched gene sets in HALLMARK collection by the high IL7R expression sample. Each line representing one particular gene set with unique color, *p* < 0.05 were considered significant. **(B)** The enriched gene sets in HALLMARK by samples with low IL7R expression, *p* < 0.05.

Due to the significant difference in the expression of IL7R in lung adenocarcinoma and normal tissues, we conducted a GSEA functional enrichment analysis of the KEGG pathway for IL7R, and the results confirmed that when IL7R expression was elevated, the KEGG pathway was mainly enriched in immune-related activities, including chemokine signaling pathway, natural killer cell-mediated cytotoxicity, B cell receptor signaling pathway, cytokine-cytokine receptor interaction, leukocyte transendothelial migration, and T cell receptor signaling pathway (Figure 6A). The decrease of IL7R expression was mainly concentrated in cell metabolic activities, such as glycan biosynthesis, peroxisome, clycosylphosphatidylinositol anchor (Figure 6B). These results suggested IL7R could be a potential indicator of TME status.

### IL7R Is Associated With Immune Cell Infiltration in Lung Adenocarcinoma

**FIGURE 7:**
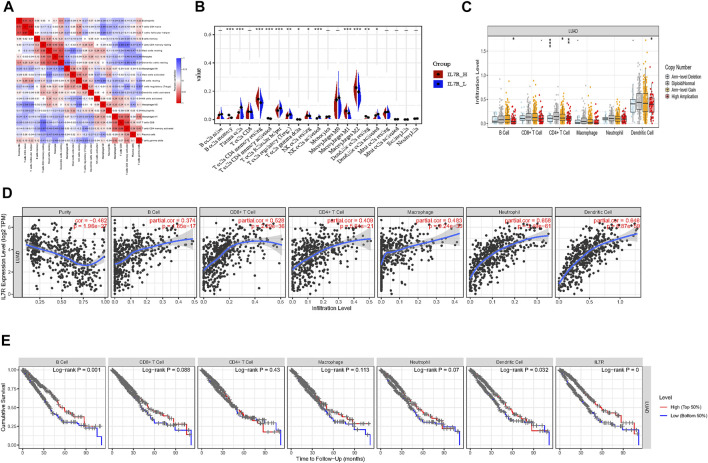
IL7R is associated with immune cell infiltration in lung adenocarcinoma. **(A)** Heatmap showing the correlation between 21 kinds of TIL and the shade of each tiny color box represented corresponding correlation value between two types of cells. **(B)** Violin plot showed the ratio differentiation of 21 kinds of immune cells between LUAD tumor samples with low or high IL7R expression, and Wilcoxon rank-sum was used for the significance test. **(C)** IL7R gene copy number variation significantly affected the infiltration levels of B cells, CD4 + T cells, and DC cells. **(D)** IL7R was negatively correlated with the tumor purity of lung adenocarcinoma and positively correlated with the expression of B cells, CD8+T cells, CD4+T cells, macrophages, monocytes, and DC cells. **(E)** Survival analysis showed the overall survival of patients with lung adenocarcinoma was positively correlated with the expression of B cells, DC cells, and IL7R.

To explore the mechanism of IL7R affecting the TME of lung adenocarcinoma, we analyzed the relationship between IL7R expression and the components of tumor-infiltrating lymphocytes in the TME of lung adenocarcinoma. The proportion of lung adenocarcinoma TIL was estimated using CIBERSORT analysis. The proportions and correlation of various TIL in TCGA lung adenocarcinoma samples were shown in Figure 7A. Grouped according to the median expression of IL7R, the infiltrating levels of 13 groups of TIL cells were found to be altered (Figure 7B). Next, the TIL ratio of TCGA lung adenocarcinoma samples was estimated by Timer algorithm, and the relationship between the changes of TIL components and the expression of IL7R was analyzed. We found the IL7R gene copy number variation significantly affected the infiltration levels of B cells, CD4 + T cells, and DC cells (Figure 7C).IL7R was negatively correlated with the tumor purity of lung adenocarcinoma and positively correlated with the expression of B cells, CD8+T cells, CD4+T cells, macrophages, monocytes, and DC cells (Figure 7D). Survival analysis showed the overall survival of patients with lung adenocarcinoma was positively correlated with the expression of B cells, DC cells, and IL7R (Figure 7E).

## Discussion

TME is composed of various types of immune cells, their secreted products (cytokines, chemokines), and extracellular matrix (Killock, 2018). Previous studies have suggested that the immune microenvironment plays an important role in the occurrence and development of primary lung cancer, and affects the treatment and prognosis of lung cancer (Almatroodi et al., 2016; Condamine et al., 2016; Bauer et al., 2017; Carrega and Ferlazzo, 2017; Janakiram et al., 2017). Studying the relationship between DEGs and the immune microenvironment, exploring the underlying mechanism may provide a new landscape for immunotherapy. In this study, we mined the expression profile data of a large number of lung adenocarcinoma samples from TCGA, evaluated the immune and stromal components of the samples, and calculated the immune/stromal scores. The immune/stromal score was determined to be significantly correlated with the molecular subtypes, clinical stage, and overall survival of lung adenocarcinoma, demonstrating that the immune microenvironment of lung adenocarcinoma is highly correlated with the clinicopathological features of lung adenocarcinoma.

Lung adenocarcinoma can be divided into three molecular subtypes, named bronchioid, squamoid, and magnoid according to its genetic pattern (Almatroodi et al., 2016; Condamine et al., 2016; Bauer et al., 2017; Carrega and Ferlazzo, 2017; Janakiram et al., 2017). These subtypes are associated with prognosis, patients with bronchioid subtype may have a superior survival and the survival was worse in magnoid tumors compared with both bronchioid and squamoid tumors (Wilkerson et al., 2012). Our result showed that both the squamoid and bronchioid subtypes had higher immune and stromal scores than the magnoid subtype. These results further suggested that high immune/stromal scores may be associated with better outcomes in patients with bronchioid subtypes. With the increase of tumor diameter, the immune/stromal scores decreased, suggesting that the immune microenvironment components of LUAD may have inhibitory effects on the formation and proliferation of tumors. Survival analysis showed that patients with high immune/stromal scores had better OS and PFS than those with low scores, indicating that the immune/stromal score of lung adenocarcinoma is a good prognostic marker for lung adenocarcinoma. Therefore, we believed the TME of lung adenocarcinoma played an important role in the development of tumors and the prognosis of patients.

To identify immune-related genes that play crucial roles in the TME of lung adenocarcinoma, we screened DEGs according to the immune/stromal scores. A total of 525 DEGs were screened by intersection analysis, among which 18 genes were up-regulated and 507 genes were down-regulated. GO enrichment functional analysis was performed on all down-regulated genes, and we found that the functions of DEGs were mainly focused on the immune response. Then, we identified 35 genes with important functions among the DEGs by constructing PPI networks. These hub genes showed significant roles related to immune response, indicating that these 35 genes play an important role in the TME of LUAD.

To search for prognostic genes for LUAD, we performed Cox survival analysis on lung adenocarcinoma data of TCGA and GSE68465 datasets. There were 294 prognostic genes in the TCGA dataset and 59 prognostic genes in the GSE68465 dataset. By intersection analysis of the prognostic genes from the above two datasets, 43 prognostic genes were screened out. These prognostic genes were found to be associated with T cell synthesis, MHCII receptor function, antigen presentation, and other immune responses. We compared the previous 35 immune microenvironment-related genes with 43 prognostic genes and finally obtained six overlapping genes. We downloaded the list of immune gene symbols for lung adenocarcinoma from the Immport website for comparison, and four of them were determined to be immunity-related genes. Therefore, we suggest that these four genes are key genes that play important roles in the TME of LUAD and influence the prognosis of patients. Cox survival analysis of these four genes revealed that only IL7R was an independent prognostic factor for OS in LUAD patients.

TCGA and Oncomine database analysis showed that the expression of IL7R was significantly higher in normal lung tissues than in LUAD tissues, this result was further verified by tissue samples and cell lines. The IL7R expression was negatively correlated with tumor size and positively correlated with overall survival and progression-free survival in LUAD patients. In order to clarify the functional mechanism of IL7R in LUAD, we performed GSEA functional enrichment analysis on IL7R. The results showed that the function of IL7R was related to the B cell receptor, T cell receptor, NK cell regulation of cytotoxicity, leukocyte chemotaxis, and other immune functions. Therefore, we speculate that IL7R may be involved in the regulation of lymphocyte infiltration in the TME, thus influencing the progression of lung adenocarcinoma.

TIL is an important component of TME, mainly consisting of T lymphocytes, B lymphocytes, natural killer (NK) cells, and other immune cells. These immune lymphocyte phenotypes can promote or inhibit the development of tumors (Rosenberg et al., 1986). TIL distribution characteristics can be used to predict the prognosis of cancer patients and the efficacy of immunotherapy (Keren et al., 2018). By the CIBOSORT analysis, we found that there were significant differences in the proportion of TIL among LUAD patients with different IL7R expression levels, which proved that IL7R could regulate TIL infiltration status. Based on the infiltration levels of TIL in groups with different IL7R expression levels, we found 12 groups of TILs had significant changes in the infiltration levels. Next, we evaluated the correlation between the IL7R expression and the infiltration level of TIL using the Timer algorithm. The result indicated that the expression of IL7R was positively correlated with all six types of TILs, and the gene copy number variation of IL7R has significantly affected the infiltration level of B cells, CD4 + T cells, and DC cells. Survival analysis showed only B cells, DC cells, and IL7R were significantly associated with the OS of LUAD patients. Above all, we believe IL7R inhibits the progression of lung adenocarcinoma by modulating the infiltration levels of B cells, DC cells, and CD4 + T cells, thus affecting the survival of patients.

IL7R is a protein-coding gene, which encodes the receptor of interleukin 7 (IL7) (Mazzucchelli and Durum, 2007). It is critical for the development of T cells and B cells, the survival of juvenile T cells, and the maintenance of memory T cells (Clark et al., 2014; Leung et al., 2019). It is necessary for the development and maintenance of innate lymphoid cells, also for the generation and development of the lymphoid structural barriers (Seddon et al., 2002). IL7R deficiency is associated with severe combined immunodeficiency (SCID) (Puel et al., 1998; Roifman et al., 2000). In our study, we found that IL7R was significantly associated with infiltration of B cell, CD4^+^ T cell, DC cell, and it also affected the prognosis of patients with LUAD. The infiltrating B lymphocyte is an important part of tertiary lymphoid structures (TLSs), which is an ectopic lymphoid organ that forms in non-lymphoid tissues during chronic inflammation and tumor progression (Sautès-Fridman et al., 2019). B cell infiltration and the formation of TLSs were found to be positively correlated with the immunotherapy response in patients with different tumor types. It is suggested that infiltrating B lymphocytes play an important role in the treatment of ICB and predict the prognosis (Cabrita et al., 2020; Helmink et al., 2020; Petitprez et al., 2020).CD4+ T cells are mainly expressed as helper T cells, and assist in the activation of other cells. Cytokines secreted by Th1 cells in the subsets of CD4^+^ helper T cells, especially interleukin-2 (IL-2) and IFN-γ, can activate and promote the function of CD8+T cells and NK cells (Knutson and Disis, 2005). Dendritic Cells (DC) are professional Antigen-presenting Cells (APC), which have attracted much attention in recent years. DC cells can absorb, process, and present antigens to initiate T cell-mediated immune response (Laoui et al., 2016). Above all, our results suggest that IL7R can influence the distribution of TIL, and inhibit tumor occurrence and progression by increasing the infiltration proportion of the above three TILs.

In conclusion, IL7R is a biomarker for predicting a good prognosis of lung adenocarcinoma. IL7R could also be a potential therapeutic target for the treatment of lung adenocarcinoma.

## Data Availability

The datasets presented in this study can be found in online repositories. The names of the repository/repositories and accession number(s) can be found in the article/Supplementary Material.
