# Supplementary figures and images for: IL7R Is Correlated With Immune Cell Infiltration in the Tumor Microenvironment of Lung Adenocarcinoma
Source: Front Pharmacol. 2022 Feb 21;13:857289. doi: 10.3389/fphar.2022.857289 (PMC8899515; doi:10.3389/fphar.2022.857289)

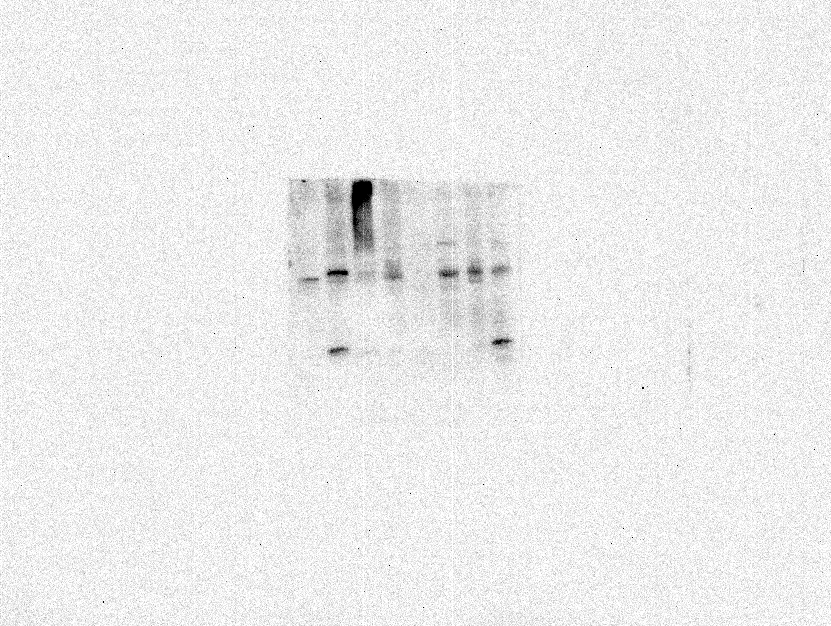

Supplement: Supplementary file 4 [file Image2.PNG]

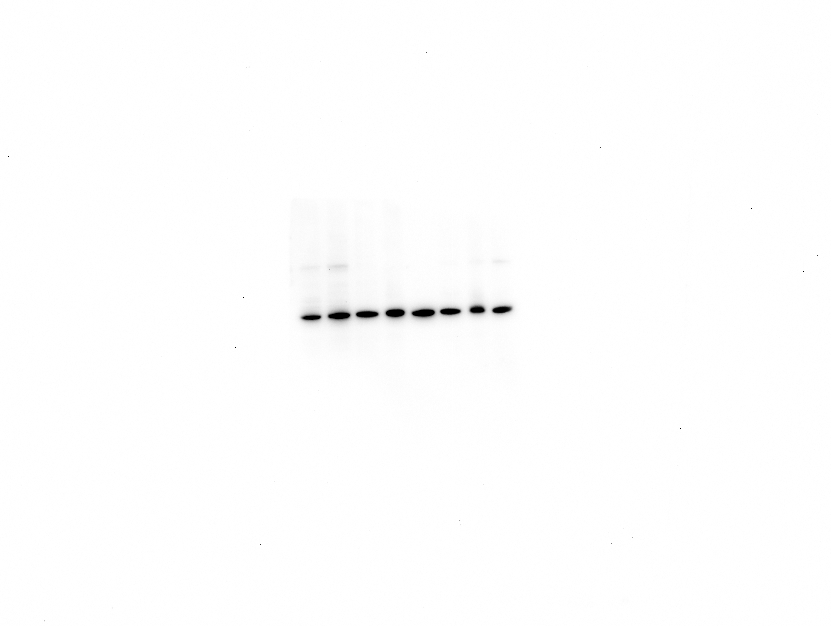

Supplement: Supplementary file 6 [file Image1.PNG]
